# P02.147. Emotions matter: sustained reductions in chronic non-structural pain after a brief, manualized emotional processing program

**DOI:** 10.1186/1472-6882-12-S1-P203

**Published:** 2012-06-12

**Authors:** H Schubiner, A Burger, M Lumley

**Affiliations:** 1 Providence Hospital, Wayne State University, Southfield, USA; 2 Cedarville University, Cedarville, USA; 3 Wayne State University, Detroit, USA

## Purpose

Current treatments for chronic pain have limited effectiveness. Although major life stressors are elevated in those with chronic pain, emotional awareness, expression, and processing have not been utilized. We evaluated treatment outcomes and predictors of outcomes of a novel emotion-oriented intervention for chronic pain.

## Methods

An initial individual session was conducted by one of the authors (HS) to assess medical conditions and review life stressors and symptom progression in order to identify linkages between stressors and symptoms. The treatment program consisted of 4 weekly small group 2-hour sessions. Components included readings, writing about emotions, mindfulness and emotional awareness exercises (on CD), and other techniques to help people identify and process emotions related to stress and pain. Homework (e.g., writing, mindfulness exercises) was assigned daily. Patients were assessed at baseline by a research team and at post-treatment and 6-month follow-up. Included instruments were the Brief Pain Inventory (BPI) and the McGill Pain Questionnaire (MPQ).

## Results

Fifty-nine adults with chronic musculoskeletal pain, primarily headaches, neck, back, and widespread pain (fibromyalgia), were included. Individuals with significant structural disease processes were excluded. Baseline demographics and characteristics were 76% women (mean age 51 years). 91% Caucasian, mean duration of pain 8.8 years, and baseline pain level 5.03 (0-10). Percent improvement was calculated for pain (% change from baseline BPI score). At post-treatment 64% had ≥ 30% improvement and 43% had ≥ 50% improvement; at 6-months 67% had ≥ 30% improvement and 53% had ≥ 50% improvement. Mean BPI scores were 5.38, 3.04, and 3.03 (p< 0.001, d=-1.27), respectively. The MPQ showed similar reductions.

## Conclusion

This high rate of improvement may surpass that of cognitive-behavioral interventions for chronic pain. An approach focusing on confronting emotional contributions to pain appears beneficial. Studies with control groups are underway to determine if targeting unresolved stress and emotions offers an advance in the treatment of chronic non-structural pain.

